# Predicting chemotherapy response using a variational autoencoder approach

**DOI:** 10.1186/s12859-021-04339-6

**Published:** 2021-09-22

**Authors:** Qi Wei, Stephen A. Ramsey

**Affiliations:** 1 School of Electrical Engineering and Computer Science, Oregon State University, Corvallis, OR USA; 2 Department of Biomedical Sciences, Oregon State University, Corvallis, OR USA

**Keywords:** Variational auto-encoder, Transcriptome, TCGA, Chemotherapy drug response classification, Cancer, Colon adenocarcinomas, Pancreatic adenocarcinoma, Bladder carcinoma, Sarcoma, Breast invasive carcinoma

## Abstract

**Background:**

Multiple studies have shown the utility of transcriptome-wide RNA-seq profiles as features for machine learning-based prediction of response to chemotherapy in cancer. While tumor transcriptome profiles are publicly available for thousands of tumors for many cancer types, a relatively modest number of tumor profiles are clinically annotated for response to chemotherapy. The paucity of labeled examples and the high dimension of the feature data limit performance for predicting therapeutic response using fully-supervised classification methods. Recently, multiple studies have established the utility of a deep neural network approach, the variational autoencoder (VAE), for generating meaningful latent features from original data. Here, we report the first study of a semi-supervised approach using VAE-encoded tumor transcriptome features and regularized gradient boosted decision trees (XGBoost) to predict chemotherapy drug response for five cancer types: colon, pancreatic, bladder, breast, and sarcoma.

**Results:**

We found: (1) VAE-encoding of the tumor transcriptome preserves the cancer type identity of the tumor, suggesting preservation of biologically relevant information; and (2) as a feature-set for supervised classification to predict response-to-chemotherapy, the unsupervised VAE encoding of the tumor’s gene expression profile leads to better area under the receiver operating characteristic curve and area under the precision-recall curve classification performance than the original gene expression profile or the PCA principal components or the ICA components of the gene expression profile, in four out of five cancer types that we tested.

**Conclusions:**

Given high-dimensional “omics” data, the VAE is a powerful tool for obtaining a nonlinear low-dimensional embedding; it yields features that retain biological patterns that distinguish between different types of cancer and that enable more accurate tumor transcriptome-based prediction of response to chemotherapy than would be possible using the original data or their principal components.

**Supplementary Information:**

The online version contains supplementary material available at 10.1186/s12859-021-04339-6.

## Introduction

### Background

Although chemotherapy is a mainstay of treatment for aggressive cancers, many agents have serious side effects [1]. Whether or not chemotherapy will provide a net benefit to a patient depends in large part on whether the malignancy responds to the treatment. Chemotherapy is often administered in cycles [2], leading to multiple opportunities where treatment appropriateness may be (re-)assessed [3]. Currently, the medical cost-benefit of chemotherapy (versus a non-pharmaceutical approach) is assessed in light of patient health status, expected therapeutic tolerance, and tumor pathological classification [4, 5]. For many cancer types, there is a broad spectrum of cases where the decision of whether or not to undergo chemotherapy is difficult [6–8]. The development of a quantitative model that could predict—based on a specific tumor’s molecular profile—whether or not the tumor will respond to chemotherapy would have significant clinical utility. Moreover, an advance in machine-learning methods for the response-to-chemotherapy prediction problem [9, 10] would have potential benefits for other prediction problems in medicine.

Tumorigenesis is driven by alterations in the somatic genome and epigenome in cancer cells [11]; however, the somatic genetic or epigenetic determinants of response to chemotherapy also affect gene expression. Studies of various cancer types have demonstrated that tumor gene expression biomarkers correlate with the probability that a tumor will respond to chemotherapy, for example, a five-protein signature in breast cancer [12], a 13-gene signature in rectal cancer [13, 14], a 63-gene signature in liver cancer [15], and a support vector machine (SVM)-based model to predict survival time in breast cancer based on a 19-gene signature [16]. The findings from such “omics” studies suggest that RNA sequencing (RNA-seq)-based transcriptome measurements of tumor samples labeled for clinical response can be used to train machine-learning classifiers for predicting response to chemotherapy. However, the accuracy of models that can be learned by fully supervised approaches is limited by the small number of available clinically labeled training cases, given that tumor transcriptome data are high-variance and high-dimensional.

For typical cancers, most available tumor transcriptomes are not labeled for chemotherapeutic response; the ratio of such unlabeled to labeled tumor datasets in the Cancer Genome Atlas (TCGA; [17]) is in the range of 10–20, depending on the cancer type. Unlabeled data are a substantial resource that could—in the context of a *semi-supervised* approach—reveal multivariate patterns that could ultimately improve predictive accuracy. Semi-supervised approaches that fuse unsupervised data reduction methods for low-dimensional embedding with supervised methods (such as decision trees) for prediction have proved beneficial in problems where large unlabeled datasets are available; for example, a principal components analysis (PCA)-XGBoost method has been previously used in finance [18], and an independent component analysis (ICA)-based method has been used to classify electroencephalographic signals [19].

### Previous applications of VAE in cancer

Multiple studies [20–23] have demonstrated the power of the variational autoencoder (VAE; [24, 25])—an unsupervised nonlinear data embedding model in which two deep neural networks are oppositely connected through a low-dimensional, probabilistic latent space—for finding useful features in high-dimensional data. In the context of cancer, VAEs have been variously used to (1) model gene expression and capture biological features using the TCGA Pan-cancer Project RNA-seq dataset [26, 27]; (2) find encodings that can be used to predict gene inactivation [28]; and (3) obtain an encoding for predicting chemotherapy resistance [29]. Way and Greene [28] explored VAE architectures for predicting gene inactivation in a pan-cancer dataset and reported biological insights obtained from the latent-space embeddings. George and Lio [29] used a VAE-based, unsupervised approach to encode tumor transcriptomes to obtain latent-space features associated with chemotherapy response. Dincer et al. [30] used a semi-supervised, VAE-lasso approach to predict drug sensitivity of cancer cells in vitro. In contrast to previous efforts to model cancer cell line drug sensitivity in vitro [30–33], in this work we focused on predicting therapeutic response in vivo, across five different cancer types (colon adenocarcinoma, pancreatic adenocarcinoma, bladder carcinoma, sarcoma, and breast invasive carcinoma). Specifically, we tested the hypothesis that a tumor transcriptome VAE would be useful for predicting response-to-chemotherapy in vivo, across multiple cancer types.

### Research objectives

We first asked to what extent VAE-encoding tumor transcriptomes would preserve characteristics that are associated with distinct cancer types. To that end, we trained a pan-cancer transcriptome VAE and used it to encode over 11k tumor transcriptomes from 33 cancer types. By comparing two-dimensional embeddings of the original tumor transcriptomes with embeddings of the VAE-encoded transcriptomes, we found (“VAE encoding preserves cancer type features” section) that the VAE preserves the clustering of tumors of the same cancer type. Next, we selected five cancer types based on sufficiency of clinical data and trained six VAE models (three architectures and two different loss functions) to encode clinically-unlabeled transcriptomes of the five cancer types. Using TCGA clinical data, we assigned a label “responded” or “progressive” to tumors where the response to chemotherapy information was available (“Obtaining a labeled tumor transcriptome dataset” section). We then used the VAE-encoded transcriptomes for the clinically-labeled tumors as feature data for predicting response to chemotherapy using gradient boosted decision trees (XGBoost; [34]), which we found to be superior to kernel SVM. Using this “semi-supervised VAE-XGBoost” approach, we investigated (“L1 loss is better than L2 loss and cross-entropy loss for this application” section) which loss function type is best for this VAE application.

**Fig. 1:**
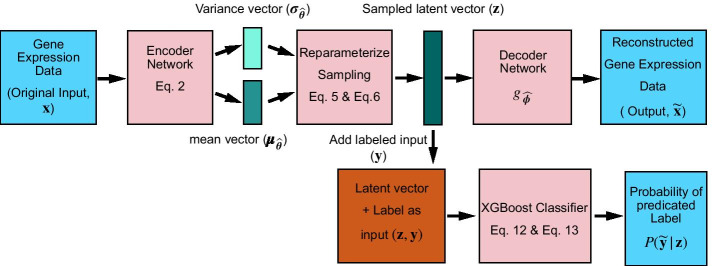
Overview of the VAE-XGBoost method that we used for predicting tumor response to chemotherapy in vivo for five different cancer types. For each tumor *t*, the encoder’s input vector ${\varvec{x}}_t$ contains the levels of the top 20% of genes by intertumoral gene expression variance. Each network has multiple fully connected dense layers (“VAE model architectures” section). The encoder outputs two vectors of configurable latent variable dimension $h \ll m$: a vector of means $\varvec{\mu }$ and a vector of standard deviations $\varvec{\sigma }$ that parameterize the multivariate normal latent-space vector ${\varvec{Z}}|{\varvec{x}}_t$ (“Variational autoencoder (VAE)” section). The sampled encoding ${\varvec{Z}}|{\varvec{x}}_t={\varvec{\boldsymbol z}}_t$ is passed to the decoding neural network (decoder), whose architecture is identical to (with inversion) that of the encoder network. The sampled latent-space vector ${\varvec{\boldsymbol z}}_t$ is passed to XGBoost for supervised classification to predict response to chemotherapy (training label ${\varvec{y}}$, prediction $\widetilde{{\varvec{y}}}$)

In the main part of this work, we focused (“Chemotherapy response classification results” section) on the question of whether and to what extent the semi-supervised VAE-XGBoost (our new method, Fig. 1) approach would improve performance for transcriptome-based prediction of response to chemotherapy, versus a fully-supervised approach or versus alternative semi-supervised approaches using PCA or ICA transcriptome encodings. We further investigated the relative importance of these approaches through the lens of XGBoost feature importance (“PCA & VAE feature importance scores, for COAD” section). We carried out these analyses using a comprehensive, five-cancer set of labeled tumor transcriptomes and obtained unbiased classification performance measurements using cross-validation.

## Results

### VAE encoding preserves cancer type features

**Fig. 2:**
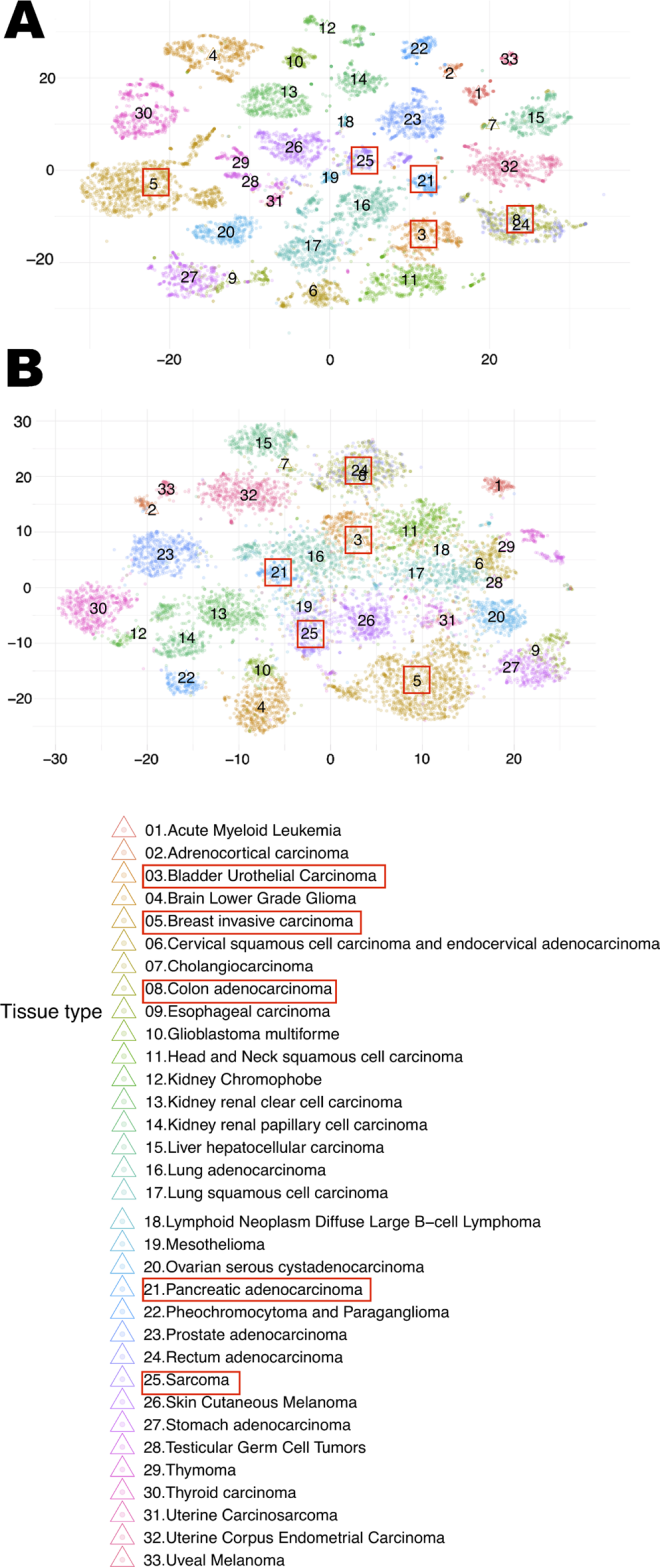
Two-dimensional embedding of the 11,057 tumor transcriptomes based on *t*-SNE. Each mark represents a transcriptome, with color representing the cancer type. **A** Original gene expression data of the top-20% highest-variance genes. **B** VAE compressed gene expression data. Red rectangles denote the five cancer types selected for chemotherapy response classification

Given reports [35, 36] that unsupervised embeddings can be used to visualize the grouping of cancer types based on high-dimensional molecular tumor data, using unsupervised methods, we investigated the extent to which VAE encoding of tumor transcriptomes preserves data-space features that determine cancer type-specific groupings. In order to do so, we obtained RNA-seq transcriptome data from the TCGA data portal for 11,057 tumors labeled for 33 different cancer types (Figs. 2, Additional file 1: S2, S3). As a baseline visualization, we generated a two-dimensional embedding of the 11,057 tumor samples by applying *t*-distributed stochastic neighbor embedding (*t*-SNE) to the expression levels of the the top-20% highest-variance genes (threshold selected as described in “Gene expression data” section), yielding 33 clusters (Fig. 2A). Next, we trained a VAE (“Variational autoencoder (VAE), VAE model architectures” sections) with a deep architecture (VAE-1) to encode the expression levels of the highest-variance genes in each of 11,057 tumors into an equivalent number of points in a 50-dimensional latent space. An unsupervised *t*-SNE visualization (Fig. 2B) of the VAE-encoded tumor transcriptome data was remarkably similar in structure to the *t*-SNE visualization of the 13,584-dimensional original dataset (Additional file 1: Fig. S1). Additionally, we compared the clustering of the original transcriptome data with VAE-reconstructed transcriptome data by Uniform manifold approximation and projection (UMAP), and found similar results (Additional file 1: Figs. S2, S3). These analyses indicated that the VAE encoding preserves data-space features that distinguish individual cancer types.

### Obtaining a labeled tumor transcriptome dataset

Having demonstrated that the VAE can efficiently encode tumor transcriptomes while preserving features that distinguish different cancer types, and to set the stage for implementing a semi-supervised approach for predicting response to chemotherapy, we obtained a five-cancer-type tumor transcriptome dataset with a significant subset of the tumors labeled as to whether or not the patient responded to chemotherapy, as described below. We obtained transcriptomes of 2,606 tumors across five cancer types [colon adenocarcinoma (COAD), pancreatic adenocarcinoma (PAAD), bladder carcinoma (BLCA), sarcoma (SARC), and breast invasive carcinoma (BRCA); Table 1]. We selected the five cancer types based on availability of a sufficient amount of labeled data in TCGA and for 806 of the tumor transcriptomes, we generated binary labels corresponding to “responded” or “progressive”.

The ratio of responding tumors to progressive disease tumors (i.e., the class balance ratio) ranged from a low of 0.77 for pancreatic cancer to a high of 8.61 for breast cancer.

**Table 1:** Numbers of tumor samples that have clinical information available regarding response-to-chemotherapy, for each cancer type (n.b., the total number of labeled tumor samples exceeds the total number of patients because some patients had multiple tumors)

Cancer type	Total number of samples (labeled and unlabeled)	Number of labeled samples	Proportion of labeled samples	Class balance ratio (responding/progressive)
Breast invasive carcinoma (BRCA)	1217	394	0.324	8.61
Colon adenocarcinomas (COAD)	512	117	0.229	1.72
Bladder carcinoma (BLCA)	430	115	0.267	0.95
Pancreatic adenocarcinoma (PAAD)	182	115	0.632	0.77
Sarcoma (SARC)	265	65	0.245	0.82
Sum	2606	806		

Each cancer type’s TCGA abbreviation is shown in parentheses

### L1 loss is better than L2 loss and cross-entropy loss for this application

**Fig. 3:**
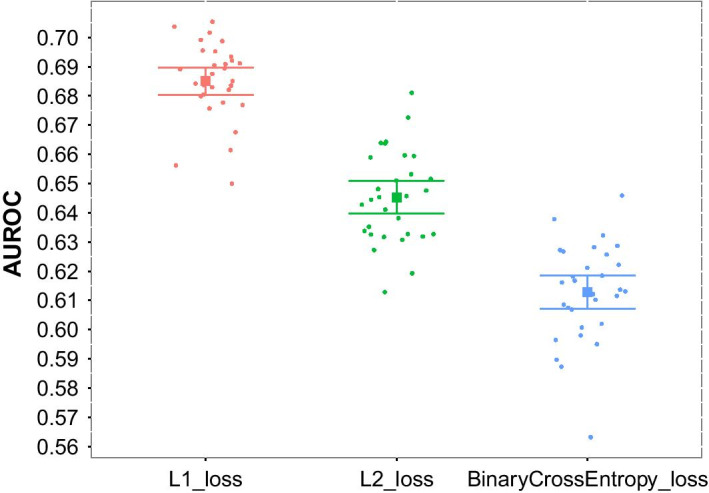
Predicting chemotherapy response using VAE-XGBoost models with different types of reconstruction loss for the VAE training. Marks represent AUROC values averaged over five different types of cancer, grouped by VAE loss function type. Squares, within-group sample mean; bars, $95\%$ confidence intervals (c.i.)

Having obtained 2,606 transcriptomes of tumors of five cancer types (with 806 of the tumors labeled by response), we next sought to determine which type of VAE reconstruction loss function—L1, L2, or binary cross entropy—would yield transcriptome encodings that are most amenable to accurate XGBoost-based prediction chemotherapy response. On the 2,606 tumor transcriptomes, we trained three sets of cancer type-specific VAEs (“VAE model architectures” section) using L1 loss, L2 loss, and binary cross-entropy loss respectively. We used the L1, L2, and binary cross-entropy VAEs to encode the 806 labeled tumor transcriptomes (the top 20% most variable genes in each cancer type, merged across the five cancers, for a total of 13,584 genes) spanning the five cancer types, yielding (for each cancer type) three feature matrices: one based on L1 loss, one based on L2 loss, and a third one based on binary cross-entropy loss. We separately evaluated the three feature matrices for XGBoost prediction of the binary response-to-chemotherapy class label. By test-set area under the receiver operating characteristic (AUROC), averaged across the five cancers, we found (Fig. 3) that the features that were generated by the L1 VAEs led to $6.2\%$ better ($p < 10^{-9}$, Welch’s *t*-test) classification performance than the features generated by the L2 VAEs, $11.7\%$ better ($p < 10^{-9}$, Welch’s *t*-test) classification performance than the features generated by the binary cross-entropy VAEs and thus, for all subsequent analyses, we used VAEs trained with L1 loss.

### Chemotherapy response classification results

Having selected L1 reconstruction loss for training VAEs to encode tumor transcriptomes for predicting response-to-chemotherapy, we developed a semi-supervised approach based on VAE encoding of the tumor transcriptome, for predicting chemotherapy response. In brief, our approach consisted of three steps:

1. Training a VAE to encode clinically *unlabeled* tumor transcriptomes (for the top 20% most variable genes) for a single cancer type, into a low-dimensional space (“VAE model architectures” section).
2. Using that VAE to obtain latent-space encodings for the tumor transcriptomes that are labeled for a relevant clinical endpoint (in this work, response to chemotherapy).
3. Training and testing a supervised classifier for predicting chemotherapy response.

Because some cancer types benefited from a deeper VAE network architecture than others for effective encoding, we used three different VAE architectures for learning features for predicting chemotherapy response in the context of three subsets of cancer types (VAE-1 for breast and pancreatic; VAE-2 for colon; and VAE-3 for bladder and sarcoma; Table 5). For each VAE architecture, our approach was to use all of the data from the five-cancer set of 2,606 unlabeled tumors for VAE training, but for *predicting* chemotherapy response for a given cancer type, we used encodings from the VAE architecture that corresponds to the cancer type (Table 5).

To select the supervised classification algorithm for step (3) above, we used an empirical approach, comparing the AUROC performance of XGBoost, kernel SVM, and *k*-nearest neighbors for predicting sarcoma response to chemotherapy with features based on VAE-3 encodings (semi-supervised) or expression levels of individual genes (fully-supervised). We found (Additional file 1: Fig. S4) XGBoost to be superior to kernel SVM and *k*-nearest neighbors (KNN), in both semi-supervised and fully supervised analyses, and thus we chose XGBoost as the classification algorithm for subsequent analyses.

To address the primary question of to what extent a VAE-based, semi-supervised (VAE-XGBoost) approach could advance the state-of-the-art for transcriptome-based prediction of chemotherapy response, we sought to compare VAE-XGBoost’s performance to that of three alternative approaches: (1) a semi-supervised approach using a regular auto-encoder (AE) with the same architecture as the VAE; (2) a fully supervised approach directly using the transcriptome data; and (3) a semi-supervised approach based on a traditional dimensional reduction technique (either principal component analysis, PCA; or independent component analysis, ICA).

#### VAE-XGBoost versus AE-XGBoost

To address alternative approach (1) (“traditional auto-encoder”), we compared the performance of VAE-XGBoost to that of a model consisting of a regular auto-encoder combined with XGBoost (“AE-XGBoost”; Table 2 and Additional file 1: Figs. S5–S6). For this four-cancer analysis, we used the VAE-1 architecture for BRCA and PAAD, which was the same network that we used in the *t*-SNE analysis and the VAE-3 architecture for BLCA and SARC (Table 5). We measured performance using test-set AUROC and AUPRC using five-fold cross-validation. We found (Table 2) that VAE-XGBoost outperformed AE-XGBoost by an average AUROC increase of 14.5% and an average AUPRC increase of 16.3% over the four-cancer average (breast, pancreatic, bladder, and sarcoma), with ($p < 10^{-9}$, Welch’s *t*-test) classification performance. Thus, we used the VAE for neural network-based unsupervised embeddings, for subsequent analyses.

#### VAE-XGBoost versus fully-supervised XGBoost

To address alternative approach (2) (“fully supervised”), we empirically compared the performance of the VAE-XGBoost method to a fully supervised model in which we applied XGBoost directly to the tumor expression levels of the top 20% most variable genes (13,584 genes) as feature data. For five out of five cancer types (breast, colon, pancreatic, bladder, and sarcoma), in terms of test-set AUROC, the VAE-XGBoost approach outperformed the fully-supervised XGBoost approach (Additional file 1: Fig. S7), by Welch’s *t*-test (Table 3). In terms of test-set AUPRC, for four out of five cancer types (breast, colon, pancreatic, and bladder), the VAE-XGBoost approach outperformed the fully-supervised approach of applying XGBoost directly to the expression levels of the tumors’ top 20% most variable genes (Additional file 1: Fig. S8), by Welch’s *t*-test (Table 4); for SARC, the semi-supervised VAE-XGBoost and fully-supervised models’ performances were statistically indistinguishable.

#### VAE-XGBoost versus PCA-XGBoost and ICA-XGBoost

To address alternative approach (3), we empirically compared VAE-XGBoost to models in which PCA or ICA components were used as XGBoost features (i.e., “PCA-XGBoost” and “ICA-XGBoost”). We aimed to empirically study prediction performance of these models for each of the five cancer types separately, using the set of cancer type-specific labeled tumors (806 labeled tumors in all). For four out of five cancer types (bladder, breast, pancreatic, and sarcoma), in terms of AUROC the semi-supervised VAE-XGBoost method significantly outperformed the semi-supervised PCA-XGBoost method (Table 3 and Additional file 1: Fig. S7). Additionally, for three out of five cancer types (breast, colon, and pancreatic), the semi-supervised VAE-XGBoost method significantly outperformed the semi-supervised ICA-XGBoost method (Table 3 and Additional file 1: Fig. S7). The five-cancer average AUROC for VAE-XGBoost was 0.688, a performance gain of 6.3% over the five-cancer average AUROC for PCA-XGBoost (0.646), a gain of 6.5% over the ICA-XGBoost (0.645) and a gain of 4.5% over the fully-supervised model’s average (0.658). Notably, a single deep VAE architecture (VAE-1, which had a 50-dimensional latent space and six layers in the encoder) yielded latent-space encodings that outperformed semi-supervised PCA-XGBoost for two cancer types (breast and pancreatic); a single shallow VAE architecture (VAE-3, which had a 500-dimensional latent space and two layers in the encoder) yielded latent-space encodings that outperformed semi-supervised PCA-XGBoost for two cancer types (bladder and sarcoma).

For three out of five cancer types (breast, bladder, and pancreatic), in terms of AUPRC the semi-supervised VAE-XGBoost method significantly outperformed the semi-supervised PCA-XGBoost method (Additional file 1: Fig. S8 and Table 4). Additionally, for three out of five cancer types (breast, colon, and pancreatic), the semi-supervised VAE-XGBoost method significantly outperformed the semi-supervised ICA-XGBoost method (Additional file 1: Fig. S8 and Table 4). The five-cancer average AUPRC for VAE-XGBoost was 0.441, a performance gain of 9.1% over the five-cancer average AUPRC for PCA-XGBoost (0.403), a gain of 8.2% over the ICA-XGBoost (0.406), and a gain of 8.5% over the fully-supervised model’s average (0.405).

**Table 2:** Comparison of chemotherapy response prediction performance for XGBoost models trained with VAE-derived features versus autoencoder (AE)-derived features, for three cancer types (BRCA, BLCA, and PAAD)

Cancer type	Mean				*p* (Welch’s *t*-test)	
	AUROC		AUPRC		AUROC	AUPRC
	VAE	AE	VAE	AE	VAE versus AE	VAE versus AE
BRCA	**0**.**674**	0.575	**0**.**192**	0.137	$1.61 \times 10^{-15}$	$5.38 \times 10^{-10}$
PAAD	**0**.**738**	0.660	**0**.**764**	0.695	$3.46 \times 10^{-10}$	$6.72 \times 10^{-7}$
BLCA	**0**.**659**	0.573	**0**.**649**	0.577	$7.97 \times 10^{-12}$	$1.23 \times 10^{-7}$
SARC	**0**.**704**	0.611	**0**.**736**	0.654	$2.78 \times 10^{-7}$	$1.75 \times 10^{-6}$

The *p* values are for row-wise difference of means tests for the two columns under “AUROC” and for the two columns under “AUPRC”, respectively. For each cancer type (row), the highest mean AUROC performance is shown in boldface

**Table 3:** Comparison of chemotherapy response prediction performance (AUROC) for XGBoost models trained with original transcriptome data (“Raw data”) or transcriptome data encoded with PCA, ICA, or VAE. This analysis was carried out across five cancers (BRCA, COAD, BLCA, PAAD, and SARC)

Cancer type	AUROC (mean)				*p* (Welch’s *t*-test)		
	VAE	Raw data	PCA	ICA	VAE versus Raw data	VAE versus PCA	VAE versus ICA
BRCA	**0**.**674**	0.649	0.614	0.609	$8.07 \times 10^{-4}$	$3.80 \times 10^{-12}$	$3.39 \times 10^{-14}$
PAAD	**0**.**738**	0.694	0.710	0.685	$6.99 \times 10^{-6}$	$5.04 \times 10^{-3}$	$2.41 \times 10^{-6}$
COAD	0.707	0.674	**0**.**726**	0.689	$1.19 \times 10^{-3}$	$2.81 \times 10^{-2}$	$5.65 \times 10^{-2}$
BLCA	**0**.**659**	0.626	0.593	0.650	$7.81 \times 10^{-5}$	$4.27 \times 10^{-9}$	$2.61 \times 10^{-1}$
SARC	**0**.**704**	0.679	0.682	0.701	$3.49 \times 10^{-2}$	$2.91 \times 10^{-2}$	$8.64 \times 10^{-1}$

The *p* values are for row-wise difference of means tests for the indicated pairs of sample groups (columns). For each cancer type (row), the highest mean AUROC performance is shown in boldface

**Table 4:** Comparison of chemotherapy response prediction performance (AUPRC) for XGBoost models trained with original transcriptome data (“Raw data”) or transcriptome data encoded with PCA, ICA, or VAE. This analysis was carried out across five cancers (BRCA, COAD, BLCA, PAAD, and SARC)

	AUPRC (mean)				*p* (Welch’s *t*-test)		
Cancer type	VAE	Raw data	PCA	ICA	VAE versus Raw data	VAE versus PCA	VAE versus ICA
BRCA	**0**.**192**	0.157	0.145	0.150	$4.21 \times 10^{-6}$	$7.42 \times 10^{-10}$	$5.01 \times 10^{-8}$
PAAD	**0**.**764**	0.729	0.746	0.713	$3.38 \times 10^{-4}$	$9.12 \times 10^{-2}$	$2.02 \times 10^{-6}$
COAD	**0**.**593**	0.535	0.579	0.545	$6.52 \times 10^{-4}$	$3.91 \times 10^{-1}$	$1.27 \times 10^{-3}$
BLCA	0.649	0.623	0.587	**0**.**654**	$4.30 \times 10^{-2}$	$1.60 \times 10^{-7}$	$6.13 \times 10^{-1}$
SARC	**0**.**736**	0.713	0.714	0.729	$6.15 \times 10^{-2}$	$1.61 \times 10^{-1}$	$5.96 \times 10^{-1}$

The *p* values are for row-wise difference of means tests for the indicated pairs of sample groups (columns). For each cancer type (row), the highest mean AUPRC performance is shown in boldface

### PCA & VAE feature importance scores, for COAD

**Fig. 4:**
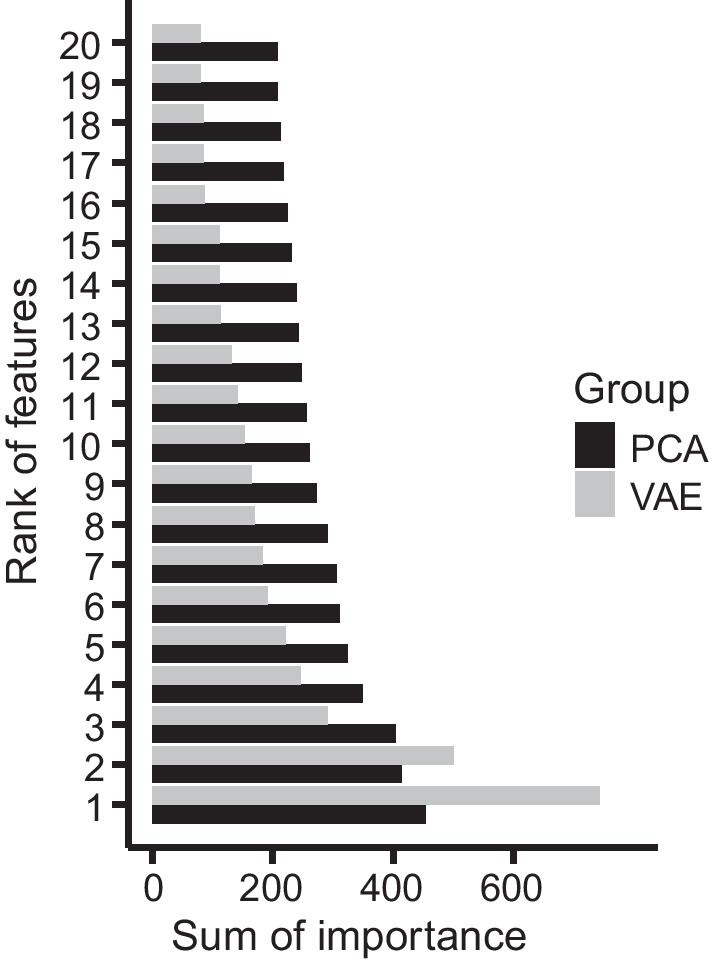
Comparing the distributions of feature importance scores for the most informative features for PCA-XGBoost and VAE-XGBoost, for predicting response to chemotherapy in colon cancer (COAD). Bars indicate the sum (over 30 replications) of XGBoost feature importance scores. “Group” indicates the low-dimensional embedding method used (VAE or PCA). Bars separately ordered from highest to lowest (only top 20 most important features shown)

Having established that the semi-supervised VAE-XGBoost outperforms the semi-supervised PCA-XGBoost approach for tumor transcriptome-based prediction of chemotherapy response for four out of five cancer types, we sought to understand the basis for the higher performance of PCA-XGBoost over VAE-XGBoost on the fifth cancer type, colon adenocarcinoma (COAD). Specifically, we investigated whether the strong performance of PCA-XGBoost on COAD is attributable to differences in the distributions of XGBoost feature importance scores of the PCA features versus VAE latent-space features. We found that the distribution of feature importance scores (as a function of rank) was more sharply peaked at lowest-ranked features in the VAE than in the PCA (Fig. 4), suggesting that the performance gain with PCA reflects a broader spectrum of informative features for that particular cancer type.

## Discussion

As far as we are aware, this work is the first report of a multi-cancer investigation of the potential for a VAE-based, semi-supervised approach for predicting in vivo chemotherapy response from the tumor transcriptome. Across the five cancer types that we studied, the ratio of responding tumors to progressive disease tumors ranged from a low of 0.77 for pancreatic cancer to a high of 8.61 for breast cancer, reflecting a broad range of resistances to standard-of-care chemotherapy. Our results clearly demonstrate the utility of the VAE for compressing high-dimensional data to a continuous, low-dimensional latent space while retaining features that are essential for distinguishing different cancer types and for predicting response to chemotherapy. Nevertheless, three limitations of this work bear noting.

The first limitation concerns the type(s) of tumor “omics” data from which features are derived for the predictive model. While in this work we focused on tumor transcriptome data which can be measured with high precision over a wide dynamic range of transcript abundances by RNA-seq, we note that TCGA datasets of tumor somatic mutations and copy number alteration events are also available [17]. Given the voluminous literature on the use of tumor somatic genomic data for precision cancer diagnosis [37–39], tumor DNA datasets are fertile ground for developing a semi-supervised, multi-omics model for predicting response to chemotherapy.

Second, for decision tree-based response-to-chemotherapy prediction, the performance of VAE-encoded transcriptome features is somewhat sensitive to the type of normalization used for the gene expression levels (data not shown). We explored various published normalization methods for the RNA-seq data including standardization of log counts and using FPKM; we ultimately chose min-max-normalized $\hbox {log}_2$ total-count-normalized counts for the gene expression levels to be used to derive features. However, there are additional transcript quantification methods [40] that could be explored in the context of finding optimal tumor transcriptome VAE encodings for precision oncology. A similar comment applies to the specific form of the reconstruction loss function: in our analysis, features from the VAE trained with L1 loss clearly (across five cancers) outperformed those from the VAE trained with L2 or cross-entropy loss, and thus, consistent with Way and Greene [28], we used L1 loss for the VAE that we used to address the main question of this work as well as the pan-cancer *t*-SNE analysis.

The third limitation relates to the VAE architecture. While it is promising that a single deep VAE architecture (VAE-1, with a 50-dimensional latent space and six fully-connected layers) yielded features that outperformed PCA and the original RNA-seq feature data for two different cancer types (breast and pancreatic), for the other three cancer types, it was necessary to use shallower (two-layer) VAE architectures with bigger latent space dimensions (400 and 500, respectively). Of the five cancer types that we studied, colon cancer and sarcoma had the lowest proportions of labeled samples (0.229 and 0.245 respectively; see Table 1). Our findings suggest that for some cancers, a deep, low-latent-dimension VAE architecture yields optimal features for predicting response, while for other cancers, a shallow, medium-sized-latent-dimension VAE architecture is more effective. Hu and Greene [41], based on a study employing single-cell transcriptome profiling, noted substantial performance differences with hyperparameter tuning on VAE architectures; they further noted that in terms of the robustness of performance with respect to hyperparameter variation, a base VAE with two layers was better than a deeper VAE architecture. Lakhmiri et al. [42] reported VAE architecture hyperparameter tuning as well as the training phase have a great impact on the overall precision of the network and its ability to generalize, and proposed $\Delta$-MADS, a hybrid derivative-free optimization algorithm for VAE fitting. More study with larger datasets will be required in order to determine whether a single VAE architecture could be successfully used for general-purpose tumor transcriptome feature extraction for precision oncology.

While our results show promise for the VAE in the context of a semi-supervised approach for response-to-chemotherapy prediction, for colon cancer, the VAE-XGBoost method did not outperform PCA-XGBoost (though it did outperform the fully supervised approach of XGBoost trained on the unencoded gene expression data). One possible explanation for the colon cancer-specific superior performance of PCA features over VAE features for predicting response to chemotherapy may be due to the fact that while (for COAD) feature importance for the VAE features is sharply peaked for the first few features and falls off fairly rapidly with feature rank, the PCA features have a significantly flatter distribution of relative feature importance (Fig. 4). Follow-on studies with larger datasets will be required to delineate under what circumstances transcriptome VAE encodings will prove superior to linear principal components. Multiple groups have argued [43–45] that to improve current precision oncology models, significantly expanded training datasets are needed to overcome the challenges posed by tumor heterogeneity, and that models must more broadly leverage somatic genetic and epigenetic information. We anticipate that the performance of VAE-XGBoost could improve significantly with more unlabeled and labeled tumor transcriptome data. Finally, we note a possible future extension of this work that will become feasible when larger training datasets are available: because response to chemotherapy is drug-dependent, the XGBoost classifier can easily include and use the chemotherapy drug type used for the patient (Additional file 1: Table S1) as a categorical feature.

## Conclusions

For four of the five cancer types that we studied, the semi-supervised VAE-XGBoost approach significantly outperformed a semi-supervised PCA-XGBoost approach for tumor transcriptome-based prediction of response to chemotherapy, reaching a top AUROC of 0.738 for pancreatic adenocarcinoma. For three of the five cancer types that we studied, the semi-supervised VAE-XGBoost approach significantly outperformed a semi-supervised ICA-XGBoost approach for tumor transcriptome-based prediction of response to chemotherapy. For BLCA and SARC, the semi-supervised VAE-XGBoost and ICA-XGBoost models’ performances were statistically indistinguishable. For five out of five cancer types, the semi-supervised VAE-XGBoost approach significantly outperformed a fully-supervised approach consisting of XGBoost applied to the expression levels of the top 20% most variably expressed genes. Given high-dimensional “omics” data, the VAE is a powerful tool for obtaining a nonlinear low-dimensional embedding; it yields features that retain biological patterns that distinguish between different types of cancer and that enable more accurate tumor transcriptome-based prediction of response to chemotherapy than would be possible using the original data or their principal components.

## Methods

We carried out all data processing and machine-learning tasks on a Dell XPS 8700 workstation equipped with Nvidia Titan RTX GPU and running the Ubuntu GNU/Linux operating system version 16.04. All of the analysis code that we implemented was executed in Python version 3.5.5 except that we used R version 3.3.3 for statistical analysis of AUROC and AUPRC values (“Area Under ROC Curve (AUROC), Area Under the precision-recall Curve (AUPRC)” sections), gene-level MAD calculations (“Gene expression data” section) and plotting (“Lower-dimensional embedding” section). We carried out all statistical tests using the R computing environment (version 3.3.3) and using the R software package stats version 3.4.4.

### Gene expression data

From the Xena data portal [46], we obtained TCGA Level 3 tumor RNA-seq transcriptome data of 33 cancer types (totaling 11, 057 tumors) and, for the response-to-chemotherapy prediction problem, five cancer types [colon adenocarcinomas (COAD), pancreatic adenocarcinoma (PAAD), bladder carcinoma (BLCA), sarcoma (SARC), and breast invasive carcinoma (BRCA)] totaling 2,606 tumors. We selected the five cancer types based on two criteria: (1) a sufficient number (at least 65) of paired tumor-transcriptome and clinical data samples available for the cancer type; and (2) a sufficient number (at least 180) of tumor transcriptome samples available (regardless of the clinical data availability) for the cancer type. We obtained both the RNA-seq (gene-level) total-read-count-normalized $\hbox {log}_2 (1+C)$ read counts and normalized (fragments per kilobase of transcript per million mapped reads, FPKM [47]) expression data for for 60,483 human genes. To focus the machine-learning on the portion of the tumor transcriptome that had the most variation from tumor to tumor, we identified the top 20% most variable genes as measured by the median absolute deviation (MAD) across tumors, of gene expression in terms of FPKM (we used FPKM for this purpose in order to mitigate bias due to read length and tumor-specific depth of sequencing) based on our preliminary results for prediction of response-to-chemotherapy for SARC, for different quantile thresholds of genes by variability of expression (Additional file 1: Fig. S4). For deriving feature-sets for XGBoost prediction directly based on transcript abundances or based on VAE- or PCA encoding, the 20% criterion applied to each of the five cancer types yielded a set of 13,584 genes. We computed MAD using the R package stats version 3.4.4 [48] with default parameters. After the variance-filtering step, we used the $\hbox {log}_2 (1+C)$ of total-count-normalized count values for the top-20% highest-variance genes (that were selected as described above) to obtain (or encode) feature values. We compared the performance—in terms of minimizing the VAE reconstruction loss (see “Variational autoencoder (VAE)” section)—of different feature scaling methods (no scaling, min-max normalization, and standardization [49]) and selected min-max normalization as the method that we used to rescale gene-level count data for input into the VAE.

### Lower-dimensional embedding

We computed *t*-SNE embedding components of the tumors using the function sklearn.manifold.TSNE from the python software package scikit-learn version 0.19.1 with parameters $\mathtt{init = ``pca''}$, $\mathtt{perplexity = 20}$, $\mathtt{learning\_rate=300}$, and $\mathtt{n\_iter=400}$. We computed UMAP embedding components using the function sklearn.manifold.umap.UMAP from the python software package scikit-learn version 0.19.1 with parameters $\mathtt{n\_neighbors = 50}$, $\mathtt{min\_dist = 0.3}$, and $\mathtt{metric=``euclidean''}$. For plotting the embeddings, we used the R software package ggplot2 version 3.1.1.

### Variational autoencoder (VAE)

An autoencoder is a type of model that combines “encoder” and “decoder” neural networks to learn a low-dimensional continuous data encoding from which the input signal can be approximately reconstructed [50]. A key advantage of an autoencoder is that it is unsupervised, i.e., it can be trained without labeled examples. Unlike classical autoencoders (e.g., sparse or denoising autoencoders), the variational autoencoder (VAE) is a generative probabilistic model which maps an input vector to a latent-space *random variable* (r.v.). Below, we mathematically define the VAE.

Let ${{\mathbb {T}}}$ denote the set of tumors for which the VAE is to be fit to the tumor transcriptomes (with $n \equiv |{{\mathbb {T}}}|$) and let *m* denote the number of genes for which transcript abundances are used to represent the tumor transcriptome. After min-max transformation of the tumor transcriptome measurements (“Gene expression data” section), each tumor’s transcriptome is represented as a vector ${{\varvec{x}}} \in [0,1]^m$. Let ${{\varvec{X}}}$ denote the random variable representing the population distribution from which tumor transcriptomes are sampled, and let $\varvec{\mathrm {X}} \in [0,1]^{m \times n}$ represent the composite matrix of all sampled tumor transcriptomes). We aim to learn a VAE that will comprise an encoder and decoder, with the encoder consisting of mean and variance functions ${\varvec{\mu }}: [0,1]^{m} \rightarrow {{\mathbb {R}}}^h$ and ${\varvec{\sigma }}: [0,1]^m \rightarrow {{\mathbb {R}}}_{+}^h$, respectively. Together, ${\varvec{\mu }}$ and ${\varvec{\sigma }}$ map the tumor transcriptome vector ${{\varvec{x}}}_t$ to a *h*-dimensional r.v. ${{\varvec{Z}}}|{{\varvec{x}}}_t$,

1 $$\begin{aligned} {{\varvec{Z}}}|{{\varvec{x}}}_t \sim {{\mathcal {N}}}({\varvec{\mu }}({{\varvec{x}}}_t), {\mathrm {diag}}({\varvec{\sigma }}({{\varvec{x}}}_t))), \end{aligned}$$

where $\text {diag}({\varvec{m}})$ is a matrix whose diagonal elements are the elements of the vector ${\varvec{m}}$. This equation is the same as Eq. 1 in Zemouri et al. [51]. The decoder is a function ${\varvec{g}}: {{\mathbb {R}}}^h \rightarrow [0,1]^m$ that, for an outcome ${{\varvec{Z}}}|{{\varvec{x}}}_t = {\varvec{\boldsymbol z}}_t \in {{\mathbb {R}}}^h$, maps

2 $$\begin{aligned} {\varvec{g}}: {{\varvec{z}}}_t \mapsto {\varvec{g}}({{\varvec{\boldsymbol z}}}_t) \equiv \widetilde{{\varvec{x}}}_t; \end{aligned}$$

the tilde on $\widetilde{{\varvec{x}}}_t$ denotes that it is the decoded data for the tumor transcriptome ${{\varvec{x}}}_t$. A good autoencoder should have low reconstruction error *L*, which is convenient to define in terms of the *p*-norm of the difference between the tumor transcriptome data ${{\varvec{x}}}_t$ and the reconstructed data $\widetilde{{\varvec{x}}}_t$, i.e., $||{{\varvec{x}}}_t - \widetilde{{\varvec{x}}}_t||_p^{\,p}$, where $||\;\;||_p$ denotes the *p*-norm. However, this definition of the reconstruction error is only deterministic in the context of a specific outcome ${\varvec{Z}}|{\varvec{x}}_t = {\varvec{\boldsymbol z}}_t$. Thus, it is conventional to define the reconstruction error as an *expectation value* over outcomes of ${\varvec{Z}}|{\varvec{x}}_t$,

3 $$\begin{aligned} L|({{\varvec{X}}}\!=\!{{\varvec{x}}}_t) \equiv {{\mathbb {E}}}_{ \; {\varvec{Z}}|{{\varvec{x}}}_t = {{\varvec{\boldsymbol z}}}_t}( ||{{\varvec{x}}}_t - {\varvec{g}}({{\varvec{\boldsymbol z}}}_t)||_p^{\,p}), \end{aligned}$$

where ${\mathbb {E}}_{\Omega }$ represents an expectation value over a space of outcomes $\Omega$. It should be noted the above representation of the reconstruction error is in terms of the outcome, ${{\varvec{\boldsymbol z}}}_t$, of a r.v. (${\varvec{Z}}|{{\varvec{x}}}_t$) whose distributional parameter functions ${\varvec{\mu }}$ and ${\varvec{\sigma }}$ have hyperparameters (neural network coefficients) that will be fitted. This equation is similar to Eq. 3 in Zemouri et al. [51]. Compared to the binary cross-entropy loss used in Eq. 3 in Zemouri et al. [51], our Eq. 3 used L1 loss instead (see findings from an empirical study in “L1 loss is better than L2 loss and cross-entropy loss for this application” section demonstrating the superiority of L1 over L2 or binary cross-entropy for the VAE reconstruction loss function). Because Eq. 3 is ill-suited to backpropagation, it is helpful to recast it in terms of a new random variable ${\varvec{{\mathcal {E}}}}_t$ that depends on ${{\varvec{Z}}}|{{\varvec{x}}}_t$ by

4 $$\begin{aligned} {\varvec{{\mathcal {E}}}}_t \equiv (\mathrm {diag}({\varvec{\sigma }}({{\varvec{x}}}_t)))^{-\frac{1}{2}} ({\varvec{Z}}_t|{\varvec{x}}_t - \varvec{\mu }({{\varvec{x}}}_t)). \end{aligned}$$

It follows from Eqs. 4 and 1 that ${\varvec{\mathcal E}}_t$ is standard multivariate normal,

5 $$\begin{aligned} \varvec{{\mathcal {E}}}_{t} \sim {\mathcal {N}}({\varvec{0}}, \varvec{\mathrm {I}}), \end{aligned}$$

where $\varvec{\mathrm {I}}$ is the $h \times h$ identity matrix, and thus, ${\varvec{{\mathcal {E}}}}_t$ does not depend on ${\varvec{\mu }}$, ${\varvec{\sigma }}$, or *t*. We therefore drop the subscript *t* and simply denote the rescaled latent-space random variable as $\varvec{{\mathcal {E}}}$. Solving Eq. 4 for ${\varvec{Z}}|{\varvec{x}}_t$ and applying it to Eq. 3, the reconstruction error $L|({{\varvec{X}}}\!=\!{{\varvec{x}}}_t)$ can be represented by

6 $$\begin{aligned} L|({{\varvec{X}}}\!=\!{{\varvec{x}}}_t) \;=\; {\mathbb {E}}_{{\varvec{{\mathcal {E}}}}}\left( \Big |\Big | {{\varvec{x}}}_t - {\varvec{g}}\left( {\varvec{\mu }}({{\varvec{x}}}_t) + \sqrt{\text {diag}({\varvec{\sigma }}({{\varvec{x}}}_t))} \, {\varvec{{\mathcal {E}}}} \right) \Big |\Big |_p^{\, p}\right) , \end{aligned}$$

which is amenable to backpropagation because the only r.v. in it is $\varvec{{\mathcal {E}}}$, whose distributional parameters do not depend on the neural network coefficients that we will be varying. In practice, rather than computing the multivariate integral over outcomes of $\varvec{{\mathcal {E}}}$, $L|({\varvec{X}}\!=\!{{\varvec{x}}}_t)$ is typically approximated by averaging over a limited number *J* of samples from $\varvec{{\mathcal {E}}}$,

7 $$\begin{aligned} L|({{\varvec{X}}}={{\varvec{x}}}_t) \simeq \left\langle \left( \Big |\Big | {{\varvec{x}}}_t - {\varvec{g}}\left( {\varvec{\mu }}({{\varvec{x}}}_t) + \sqrt{\text {diag}({\varvec{\sigma }}({{\varvec{x}}}_t))} \, {\varvec{\epsilon }}_j\right) \right) \Big |\Big |_p^{\, p}\Big )\right\rangle _{\!j}\!, \end{aligned}$$

where $\langle \rangle _j$ denotes average over $j \in \{1,\ldots , J\}$ and $\varvec{\epsilon }_j$ is sample *j* from $\varvec{{\mathcal {E}}}$. Following Way and Greene [28], we used a number of samples that is equivalent to the dimension of the transcriptome, i.e., $J = m$. For the case of $p=2$ (i.e., L2 norm), minimizing $L|({\varvec{X}}={{\varvec{x}}}_t)$ as defined above is equivalent to maximizing the expectation value of the log-likelihood $\log (P({\varvec{g}}({{\varvec{Z}}})={\varvec{x}}_t \mid {{\varvec{X}}}={\varvec{x}}_t))$. However, following Way and Greene [28] and consistent with empirical evidence (“L1 loss is better than L2 loss and cross-entropy loss for this application” section), for our five-cancer study of the utility of a VAE-based approach for response-to-chemotherapy prediction, as well as for the pan-cancer *t*-SNE analysis (“VAE encoding preserves cancer type features” section), we chose to use L1 reconstruction loss, i.e., $p=1$ in Eq. 3.

The reconstruction loss measures bias error, whose minimization must be balanced against the simultaneous goal of controlling variance error through regularization. In the VAE, regularization requires incentivizing (in the learning of ${\varvec{\mu }}$, $\varvec{\sigma }$, and ${\varvec{g}}$) the latent space distributions of ${\varvec{Z}}|{{\varvec{x}}}$ to be close to standard multivariate normal. This is accomplished by assigning a penalty based on the Kullback-Leibler divergence between the distribution of ${\varvec{Z}}|{{\varvec{x}}}_t$ and the target distribution $\varvec{{\mathcal {E}}}$, represented by $D_{\text {KL}}(P({{\varvec{Z}}}|{{\varvec{x}}}_t) \, || \, P({\varvec{{\mathcal {E}}}}))$. This regularization is analytically tractable [52], and for a given tumor *t* yields (Supplementary Equation, Eq. S2) the following regularization function:

8 $$\begin{aligned} D_{\text {KL}}\left( P({{\varvec{Z}}_t}|{{\varvec{x}}_t}) \; \big |\big | \;P({\varvec{{\mathcal {E}}}})\right) = ||{\varvec{\mu }}({{\varvec{x}}}_t)||_2^{\,2} + ||{\varvec{\sigma }}({\varvec{x}}_t)||_2^{\,2} - ||\log ({\varvec{\sigma }}({{\varvec{x}}}_t))||_1 - 1, \end{aligned}$$

wher﻿e $\log ({\varvec{\sigma }}_t)$ denotes an element-wise log and $||\;\;||_1$ is the L1 norm.

Fitting the VAE to $\varvec{\mathrm X}$ requires selecting $\varvec{\mu }$, $\varvec{\sigma }$, and ${{\varvec{g}}}$ from their respective function spaces; in practice, we search over functions that can be represented using a neural network for $\varvec{\mu }$ and $\varvec{\sigma }$ (parameterized by the vector ${\varvec{\theta }}$)^1^ and a neural network for the function ${{\varvec{g}}}$ (parameterized by the vector $\varvec{\phi }$). Exploring the space of functions $\varvec{\mu }_{\varvec{\theta }}$, $\varvec{\sigma }_{\varvec{\theta }}$, and ${\varvec{g}}_{\varvec{\phi }}$ corresponds to computationally searching for the vector pair $(\hat{\varvec{\theta }}, \hat{\varvec{\phi }})$ that together minimize the joint (over all tumors) sum of the tumor-specific reconstruction loss and the regularization penalty,

9 $$\begin{aligned} (\widehat{\varvec{\theta }}, \widehat{\varvec{\phi }}) = \, \underset{({\varvec{\theta }}, {\varvec{\phi }})}{\text {argmin}} \,\sum _{t \in {{\mathbb {T}}}} \left[ L|({{\varvec{X}}}={{\varvec{x}}}_t)+ D_{\text {KL}}\left( P({{\varvec{Z}}}|{\varvec{x}_t})\,\big |\big |\,P({\varvec{{\mathcal {E}}}})\right) \right] . \end{aligned}$$

Applying Eqs. 6, 7, and 8, and setting $p=1$ as discussed above, we obtain the explicit formula for fitting a VAE to $\varvec{\mathrm {X}}$,

10 $$\begin{aligned}(\widehat{\varvec{\theta }}, \widehat{\varvec{\phi }}) &= \, \mathop{\text{argmin}}_{({\varvec{\theta }}, {\varvec{\phi }})} \, \sum _{t \in {{\mathbb {T}}}} \left[ \frac{1}{J}\sum _{j=1}^J{\left( \left\| {{\varvec{x}}}_t - {\varvec{g}}_{\varvec{\phi }}\left( {\varvec{\mu }}_{\varvec{\theta }}({{\varvec{x}}}_t) + \sqrt{\text {diag}({\varvec{\sigma }}_{\varvec{\theta }}({{\varvec{x}}}_t))} \, \varvec{\epsilon }_j \right) \right\|_1\right) }\right. \nonumber \\&\quad \left.+ ||{\varvec{\mu }}_{\varvec{\theta }}({{\varvec{x}}}_t)||_2^{\,2} + ||{\varvec{\sigma }}_{\varvec{\theta }}({\varvec{x}}_t)||_2^{\,2} - ||\log ({\varvec{\sigma }}_{\varvec{\theta }}({\varvec{x}}_t))||_1 - 1\right]. \end{aligned}$$

We implemented Eq. 10 in Tensorflow version 1.4.1 with Keras version 2.1.3 as the model-level library. We solved Eq. 10 using the Adam optimization algorithm [53] (with batch normalization) from the python package keras-gpu version 2.1.3 with parameters $\mathtt{learning\_rate}=2\times 10^{-3}$, $\mathtt{beta\_1 = 0.9}$, and $\mathtt{beta\_2 = 0.999}$, to obtain $(\widehat{\varvec{\theta }}, \widehat{\varvec{\phi }})$. Then, for each tumor *t*, we used a single sample ${\varvec{Z}}|{\varvec{x}}_t = {\varvec{z}}_t$ from the distribution ${\mathcal N}(\varvec{\mu }_{\widehat{\varvec{\theta }}}({\varvec{x}}_t), \text {diag}(\varvec{\sigma }_{\widehat{\varvec{\theta }}}( {\varvec{x}}_t)))$ as the final latent-space encoding of the tumor to be used for supervised learning (“Regularized gradient boosted decision trees (XGBoost)” section).

### VAE model architectures

We trained six transcriptome-encoding VAEs based on three VAE architectures, the pan-cancer VAE architecture (for the 33-cancer unsupervised analysis, “VAE encoding preserves cancer type features” section) and three cancer type-specific VAE architectures for response-to-chemotherapy prediction (“Chemotherapy response classification results” section) (VAE-1 was used for two different cancer types, BRCA and PAAD, VAE-2 was used for COAD, and VAE-3 was used for two different cancer types, BLCA and SARC). For the pan-cancer, we used the VAE-1 model with a latent space dimension $h=50$. For the cancer type-specific VAE architectures, we again used the same number of fully-connected layers in the encoder as in the decoder (Table 5).

**Table 5:** VAE architectures used for predicting chemotherapy response (*h*, latent space dimension; “layers”, # of layers used in the encoder/decoder)

Name	Cancer types	*h*	Layers
VAE-1	BRCA, PAAD	50	Six
VAE-2	COAD	400	Two
VAE-3	BLCA, SARC	500	Two

### Labeling tumors based on response to chemotherapy

From Xena and cBioPortal [54, 55], we obtained and combined TCGA clinical data (where available) for the patients whose tumor transcriptomes we acquired (“Gene expression data” section). From Xena, we extracted the variables $\mathtt{submitter\_id.samples}$, $\mathtt{therapy\_type}$, and $\mathtt{measure\_of\_response}$; from cBioPortal, we extracted the variables $\mathtt{Sample\_ID}$, $\mathtt{Disease.Free.Status}$, and $\mathtt{Pharmaceutical.Therapy.Indicator}$. We co-analyzed the Xena- and cBioPortal-obtained clinical data to label tumors “responded” ($y=0$) or ”progressive” ($y=1$), by assigning $y = 0$ when the clinical record had $\mathtt{Complete\ response}$ or $\mathtt{partial\ response}$ in the $\mathtt{measure\_of\_response}$ column of the clinical data from Xena, or with value $\mathtt{Disease Free}$ in the $\mathtt{Disease.Free.Status}$ column of the clinical data from cBioPortal while therapy type is recorded as $\mathtt{Chemotherapy}$ in both. We assigned $y = 1$ to tumors whose clinical records had values $\mathtt{Radiographic\ progressive\ disease}$, $\mathtt{Clinical\ progressive\ disease}$, or $\mathtt{stable\ disease}$ in the Xena clinical data column $\mathtt{measure\_of\_response}$, or had value $\mathtt{Recurred}$/ $\mathtt{progressed}$ in the cBioPortal data column $\mathtt{Disease.Free.Status}$ while the $\mathtt{therapy\_type}$ is recorded as $\mathtt{Chemotherapy}$ in both files. This yielded 806 labeled tumors out of 2,606 total. A total of 39 different drugs were used to treat the 794 patients (Additional file 1: Table S1).

### Regularized gradient boosted decision trees (XGBoost)

For predicting whether or not (based on its transcriptome-derived feature-set: raw, PCA, ICA, or VAE) a tumor would respond to chemotherapy, we used XGBoost [34], an efficient implementation of regularized gradient boosted decision trees. We used the classifier function XGBClassifier from the python software package xgboost version 0.80, with gamma=0. We tuned eight hyper-parameters (Table 6) by exhaustive grid-search with five-fold cross-validation, using model_selection.GridSearchCV from scikit-learn version 0.19.1. To obtain feature importance scores, we used get_score with importance_type = cover.

**Table 6:** XGBoost classification algorithm hyperparameters and hyperparameter ranges used in grid-search tuning

Hyperparameter name	Hyperparameter description	Hyperparameter range
n_estimators	Number of trees to fit	(1, 2, 3, $\ldots$, 40)
max_depth	Maximum tree depth	(1, 2, 3, $\ldots$, 10)
learning_rate	Boosting learning rate	(0.05, 0.1, 0.2, 0.4, 0.6, 0.8)
min_child_weight	Minimum sum of instance weight needed in a child	(1, 2, 3, $\ldots$, 10)
subsample	Sub-sample ratio of the training instance	(0.1, 0.2, 0.3, $\ldots$, 1.0)
colsample_bytree	Sub-sample ratio of columns when constructing each tree	(0.1, 0.2, 0.3, $\ldots$, 1.0)
reg_alpha	Coefficient of L1 regularization for the node weights	(0, 1, 2, 3)
reg_lambda	Coefficient of L2 regularization for the node weights	(1, 2, $\ldots$, 100)

### Principal component analysis (PCA) and independent component analysis (ICA)

For PCA, we used the function decomposition.PCA (with parameters $\mathtt{svd\_solver}\!=\!\mathtt{``full''})$ and $\mathtt{n\_components}\!=\!0.9$ (90% variance, yielding 387 components) from the python package scikit-learn version 0.19.1. For plotting, we used matplotlib version 2.1.2. For ICA, we used the function decomposition.FastICA (with parameters $\mathtt{n\_components}\!=\!387$ (i.e., the same number of components as used in the PCA method) from the python package scikit-learn version 0.19.1. For plotting, we used matplotlib version 2.1.2.

### Support vector machine (SVM)

For predicting whether or not (based on its transcriptome-drived feature-set: raw or VAE) a tumor would respond to chemotherapy, we used SVM [56]. We used the classifier function SVC from the python software package sklearn.svm, with gamma = “auto”. We tuned three hyper-parameters (Table 7) by exhaustive grid-search with five-fold cross-validation, using model_selection.GridSearchCV from scikit-learn version 0.19.1.

**Table 7:** SVM classification algorithm hyperparameters and hyperparameter ranges used in grid-search tuning

Hyperparameter name	Hyperparameter description	Hyperparameter range
kernel	Kernel type to be used	(‘linear’, ‘poly’, ‘rbf’, ‘sigmoid’)
C	Regularization parameter	(5, 6, 7, $\ldots$, 50)
degree	Degree of the polynomial kernel function (‘poly’)	(1, 2, 3, $\ldots$, 20)

### K-nearest neighbors vote (KNN)

For predicting whether or not (based on its transcriptome-drived feature-set: raw or VAE) a tumor would respond to chemotherapy, we used KNN [57], an implementation based on the ${\varvec{k}}$ nearest neighbors of each query point. We used the classifier function neighbors.KNeighborsClassifier from the python software package scikit-learn. We tuned five hyper-parameters (Table 8) by exhaustive grid-search with five-fold cross-validation, using model_selection.GridSearchCV from scikit-learn version 0.19.1.

**Table 8:** KNN classification algorithm hyperparameters and hyperparameter ranges used in grid-search tuning

Hyperparameter name	Hyperparameter description	Hyperparameter range
n_neighbors	Number of neighbors to use	(1, 2, 3, $\ldots$, 20)
weights	Weight function used in prediction	(‘uniform’, ‘distance’)
algorithm	Algorithm used to compute the nearest neighbors	(‘ball_tree’, ‘kd_tree’, ‘brute’, ‘auto’)
leaf_size	Leaf sized passed to BallTree or KDTree	(1, 2, 3, $\ldots$, 20)
p	Power parameter for the Minkowski metric	(1, 2, 3, 4)

### Area under ROC curve (AUROC)

For computing the AUROC (i.e., sensitivity versus false positive error rate curve), we used the function metrics.roc_auc_score from the python software package scikit-learn version 0.19.1 with parameter average=“weighted”. We logit-transformed AUROC values before testing (using two-tailed Welch’s *t*-test). For the L1 versus L2 analysis (“L1 loss is better than L2 loss and cross-entropy loss for this application” section), we carried out 30 replications of five-fold cross-validation; within each replication, across the five folds, we obtained prediction scores for each tumor from the fold in which the tumor was in the test set, enabling us to compute an overall AUROC within each replication. For each training data set, we carried out 30 replications of five-fold cross-validation by altering the random seed used for assigning data to folds, during the cross-validation. We used the same procedure for five different cancer types (BLCA, BRCA, COAD, PAAD, SARC) as shown in the panel names of Additional file 1: Figure S7.

### Area under the precision-recall curve (AUPRC)

For computing the AUPRC, we used the function metrics.precision_recall_curve and metrics.auc from the python software package scikit-learn version 0.19.1. We logit-transformed AUPRC values before testing (using two-tailed Welch’s *t*-test). We carried out 30 replications of five-fold cross-validation; within each replication, across the five folds, we obtained prediction scores for each tumor from the fold in which the tumor was in the test set, enabling us to compute an overall AUPRC within each replication. For each training data set, we have done 30 replications of five-fold cross-validation by altering the random seed used for assign split of data during cross-validation. We have conducted the same procedure for five different cancer types (BLCA, BRCA, COAD, PAAD, SARC) as shown in the panel names of Additional file 1: Figure S8.

## Supplementary Information

**Additional file 1**. This supplementary file contains Supplementary Figures S1, S2, S3, S4, S5, S6, S7, and S8, as well as Table S1 and Supplementary Note C.

## Data Availability

Software code written for this project “VAE for chemotherapy drug response prediction” are freely available under an open-source license, platform independent, written in Python and R with CUDA and tensorflow installed, at the URL: https://github.com/ATHED/VAE_for_chemotherapy_drug_response_prediction. Supplementary data are available at *BMC Bioinformatics* online.

## Footnotes

1 Note, functions $\varvec{\mu }$ and $\varvec{\sigma }$ are just two different outputs of the encoding neural network, differing only at the final layer, and thus for simplicity of notation we represent them as having a common parameter vector $\varvec{\theta }$.
