# Supplementary material for: Predicting chemotherapy response using a variational autoencoder approach
Source: BMC Bioinformatics. 2021 Sep 22;22:453. doi: 10.1186/s12859-021-04339-6 (PMC8456615; doi:10.1186/s12859-021-04339-6)
Supplement: Supplementary file 1 — Additional file 1. This supplementary file contains Supplementary Figures S1, S2, S3, S4, S5, S6, S7, and S8, as well as Table S1 and Supplementary Note C. [file 12859_2021_4339_MOESM1_ESM.pdf]

Predicting chemotherapy response using a variational autoencoder approach: Supplementary Material

Qi Wei and Stephen A. Ramsey

July 19, 2021

A. Supplementary Figure

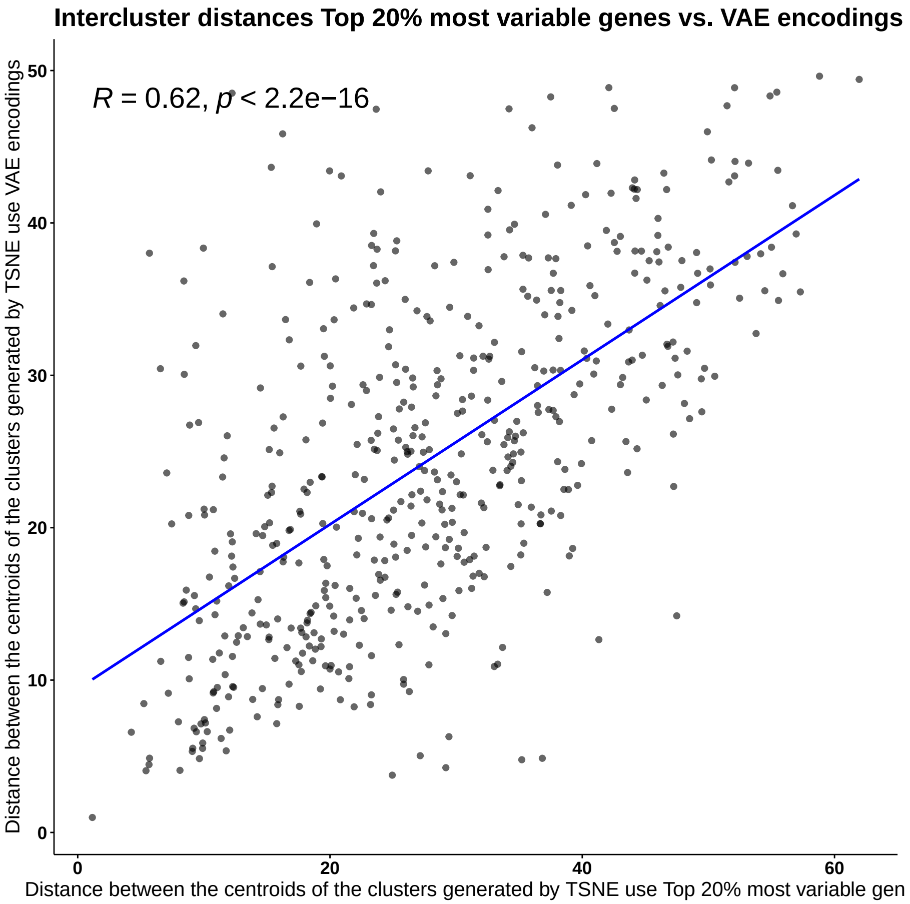

Fig. S1. Each mark corresponds to an unordered pair of cancer types (from the 33 cancer types in Fig. 2 in the main article). The horizontal axis measures the distance between the two clusters' centroids based on the *t*-SNE visualization of the tumor expression levels of the top 20% most variable genes, as in Fig. 2A (main article). The vertical axis measures the distance between the two clusters' centroids based on the *t*-SNE visualization of the VAE encoding (with latent space dimension $h = 50$) of the expression levels of the top 20% most variable genes, as in Fig. 2B (main article).

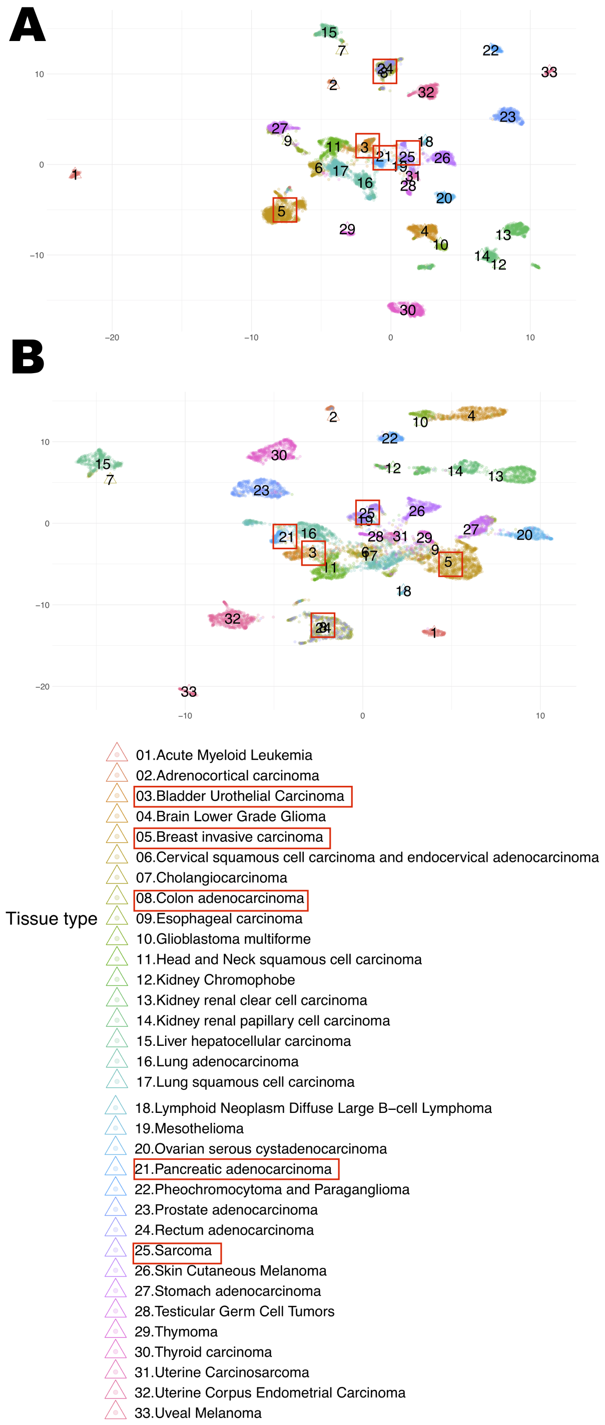

Fig. S2. Marks represent tumor transcriptomes visualized using UMAP, with colors representing cancer types. (A) Original gene expression data of the top-20% highest-variance genes. (B) Reconstructed gene expression data (same dimension as the original gene expression data) using the VAE compressed features. Red rectangles denote the five cancer types selected for chemotherapy response classification (Sec. 2.4 (main article)).

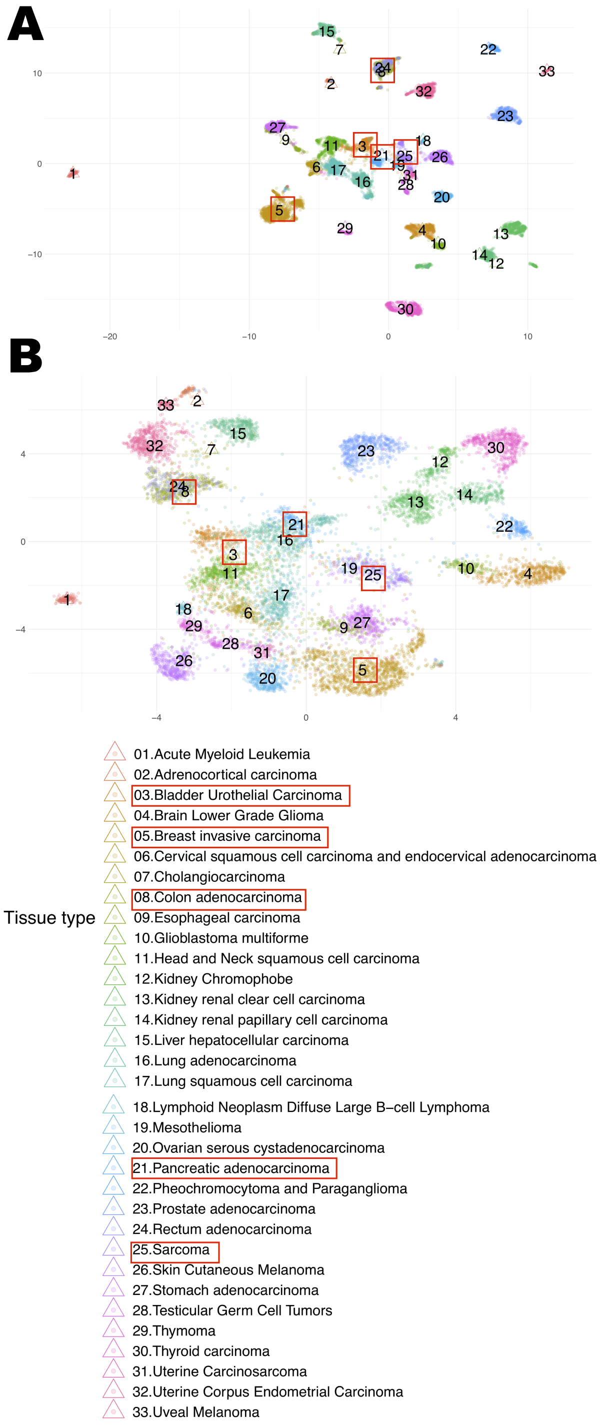

Fig. S3. Marks represent tumor transcriptomes visualized using UMAP, with colors representing cancer types. (A) Original gene expression data of the top-20% highest-variance genes. (B) VAE compressed gene expression data. Red rectangles denote the five cancer types selected for chemotherapy response classification (Sec. 2.4 (main article)).

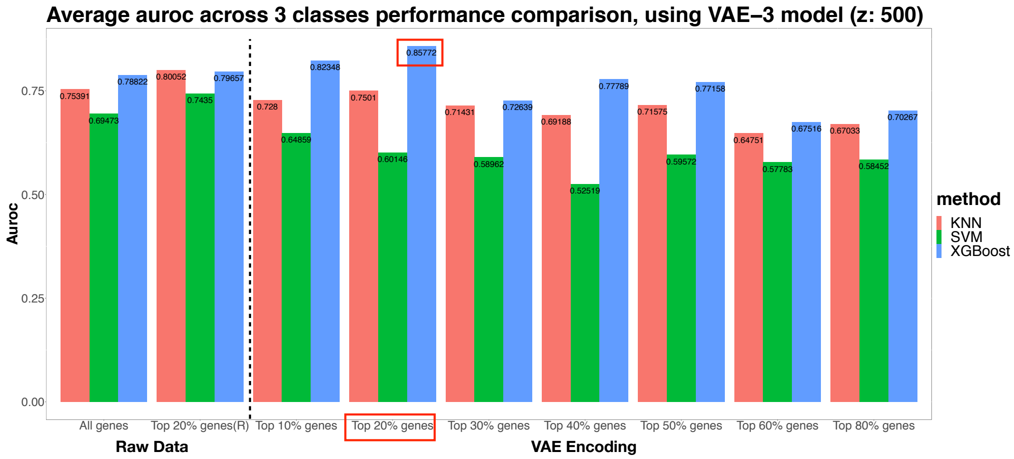

Fig. S4. Results for prediction of response-to-chemotherapy for sarcoma, for three different machine learning models (SVM: Support Vector Machine (Green, Sec. 5.8), KNN: K Nearest Neighbor (Orange, Sec. 5.9 (main article)), and XGBoost (Blue, Sec. 5.6 (main article))), for two different types of learning ("raw data", corresponding to fully suppervised learning; and "VAE encoding", corresponding to semi-supervised learning), for different quantile thresholds for selecting genes by variability of expression. XGBoost with the top 20% most variable genes has the highest average AUROC result (red rectangle). The horizontal axis represents the training dataset using different quantile thresholds of genes by variability of expression (from 10% to 80%). The vertical axis is the average AUROC across 3 classes (1. Complete Response, 2. Clinical Progressive Disease and Radiographic Progressive Disease, 3. Stable Disease).

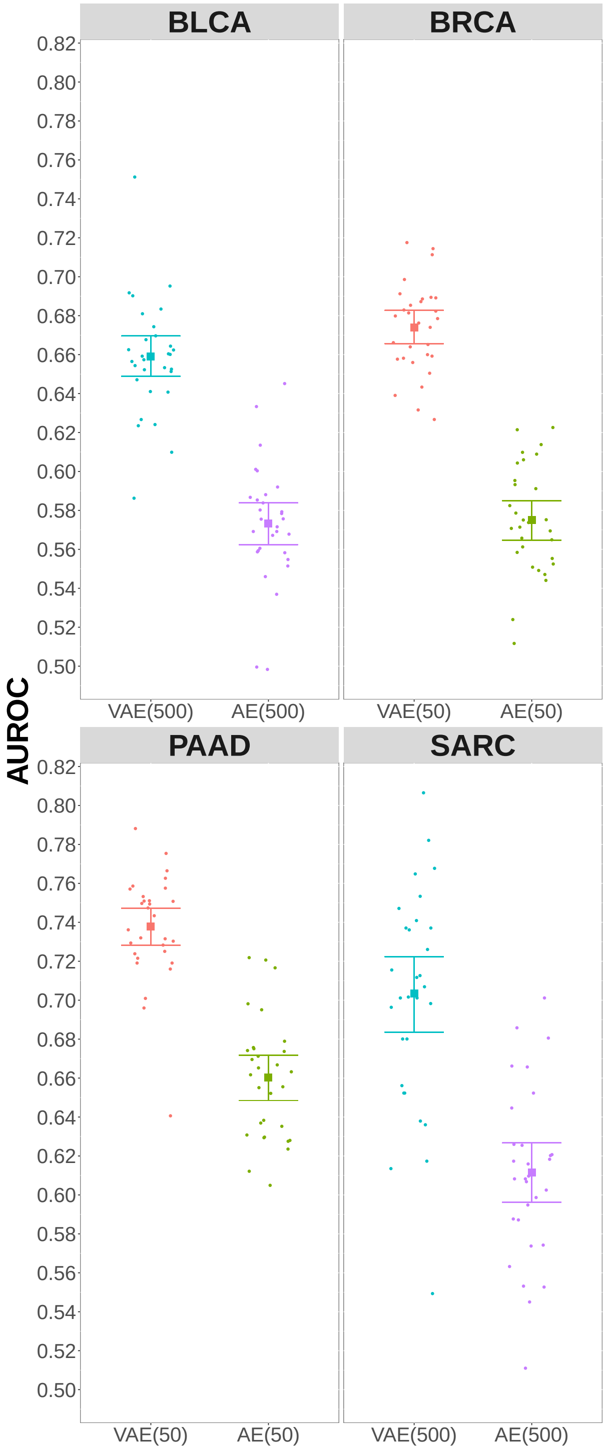

Fig. S5. Test-set AUROC performance of the two models for predicting response to chemotherapy, across five cancer types. Group abbreviations: "AE(n)", the Autoencoder-XGBoost semi-supervised method and "VAE(n)", the VAE-XGBoost semi-supervised method (n: dimension of the latent feature space). Marks correspond to individual replications of five-fold cross-validation; solid squares denote mean; bars indicate 95% c.i; colors denote the type of feature-set (Sec. 5.4 (main article)): red, `VAE-1`; green, `AE-1` (Same encoder and decoder architecture as `VAE-1`), cyan, `VAE-1`; purple, `AE-3` (Same encoder and decoder architecture as `VAE-3`).

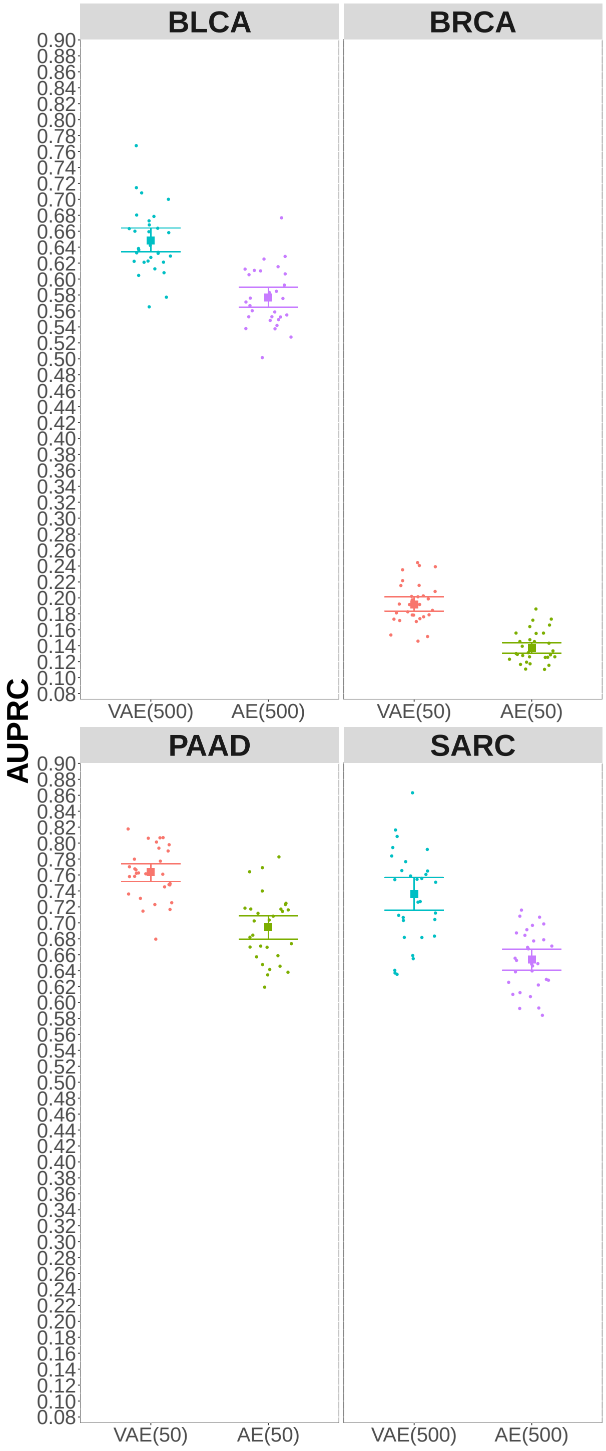

Fig. S6. Test-set AUPRC performance of the two models for predicting response to chemotherapy, across five cancer types. Group abbreviations: "AE(n)", the Autoencoder-XGBoost semi-supervised method and "VAE(n)", the VAE-XGBoost semi-supervised method (n: dimension of the latent feature space). Marks correspond to individual replications of five-fold cross-validation; solid squares denote mean; bars indicate 95% c.i; colors denote the type of feature-set (Sec. 5.4 (main article)): red, `VAE-1`; green, `AE-1` (Same encoder and decoder architecture as `VAE-1`), cyan, `VAE-1`; purple, `AE-3` (Same encoder and decoder architecture as `VAE-3`).

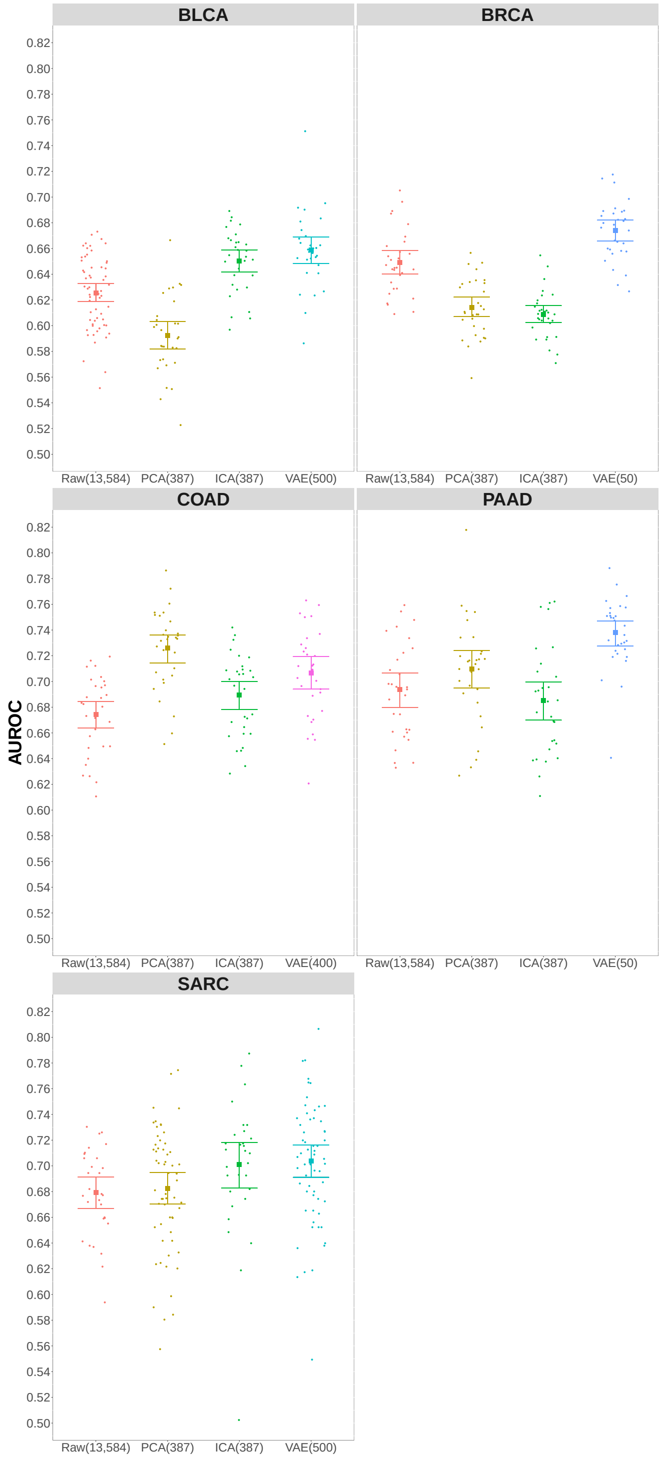

Fig. S7. Test-set AUROC performance of the three models for predicting response to chemotherapy, across five cancer types. Group abbreviations: "PCA(387)", the PCA-XGBoost semi-supervised method (387: number of principal components used as features);"ICA(387)", the ICA-XGBoost semi-supervised method (387: number of independent components used as features); "Raw(13,584)", the fully-supervised XGBoost method (13,584: number of genes used as features); and "VAE(n)", the VAE-XGBoost semi-supervised method (n: dimension of the latent feature space). Marks correspond to individual replications of five-fold cross-validation; solid squares denote mean; bars indicate 95% c.i; colors denote the type of feature-set (Sec. 5.4 (main article)): red, "Raw"; olive, "PCA"; green, "ICA"; blue, `VAE-1`; magenta, `VAE-2`; cyan, `VAE-3`).

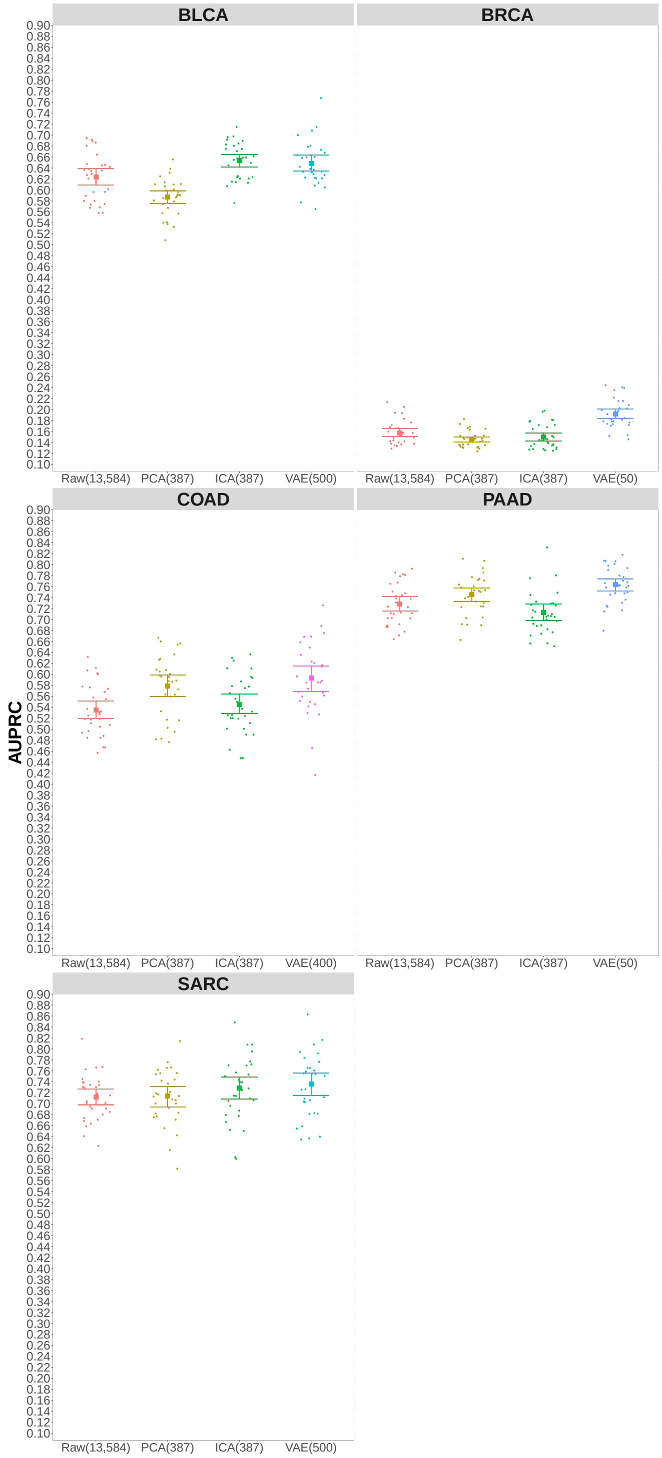

Fig. S8. Test-set AUPRC performance of the three models for predicting response to chemotherapy, across five cancer types. Group abbreviations: "PCA(387)", the PCA-XGBoost semi-supervised method (387: number of principal components used as features);"ICA(387)", the ICA-XGBoost semi-supervised method (387: number of independent components used as features); "Raw(13,584)", the fully-supervised XGBoost method (13,584: number of genes used as features); and "VAE(n)", the VAE-XGBoost semi-supervised method (n: dimension of the latent feature space). Marks correspond to individual replications of five-fold cross-validation; solid squares denote mean; bars indicate 95% c.i; colors denote the type of feature-set (Sec. 5.4 (main article)): red, "Raw"; olive, "PCA"; green, "ICA"; blue, `VAE-1`; magenta, `VAE-2`; cyan, `VAE-3`).

B. Supplementary Table

Table S1. Therapeutic agents that were used to treat patients in the TCGA clinical dataset (Hutter and Zenklusen, 2018; Goldman *et al.*, 2019) (see Sec. 5.1), for the five cancer types for which our model was trained to predict response. In some cases, the specific chemotherapeutic agent was not available in the TCGA clinical record.

cancer type	therapeutic agents
breast invasive carcinoma (BRCA)	Goserelin, Trastuzumab, Fluorouracil, Cyclophosphamide, Gemcitabine, Docetaxel, Doxorubicin, Capecitabine, Paclitaxel, Bevacizumab, Methotrexate, Vinorelbine, Etoposide, Carboplatin, Tamoxifen, Pemetrexed, Lapatinib, Everolimus, Epirubicin, Letrozole
colon adenocarcinomas (COAD)	Fluorouracil, Oxaliplatin, Irinotecan, Capecitabine, Leucovorin, Cetuximab, Regorafenib
bladder carcinoma (BLCA)	Cisplatin, Etoposide, Paclitaxel, Carboplatin, Gemcitabine, Doxorubicin, Fluorouracil, Docetaxel, Methotrexate, Ifosfamide, Vinblastine, Vorinostat, Platinum, Vinorelbine, Vandetanib
pancreatic Adenocarcinoma (PAAD)	Gemcitabine, Fluorouracil, Oxaliplatin, Irinotecan, Capecitabine, Cisplatin, Erlotinib, Paclitaxel
sarcoma (SARC)	Gemcitabine, Pazopanib, Docetaxel, Temozolomide, Cisplatin, Ifosfamide, Palbociclib, Dacarbazine, Alisertib, Doxorubicin, Carboplatin, Pemetrexed, Sorafenib, Tivozanib

C. Supplementary Equation

Using the Kullback-Leibler divergence as the measure of deviation and assuming the latent prior is iid Gaussian, the inputs are two multivariate normal distributions of dimension h, for which in general the KL divergence formula (Duchi, 2007) is written as:

$$D_{\mathrm{KL}}(p_1||p_2) = \frac{1}{2}[\log\frac{|\Sigma_2|}{|\Sigma_1|} - h + tr\{\Sigma_2^{-1}\Sigma_1\} + (\mu_2-\mu_1)^T\Sigma_2^{-1}(\mu_2-\mu_1)], \tag{S1}$$

where $p_1 = \mathcal{N}(\mu_1, \Sigma_1)$ and $p_2 = \mathcal{N}(\mu_2, \Sigma_2)$. In VAE model, we have $p_1 = P(\boldsymbol{Z}|\boldsymbol{x})$ and $p_2 = P(\boldsymbol{\mathcal{E}}))$ and Equation S1 can be written as:

$$\begin{aligned} D_{KL}(P(\boldsymbol{Z}|\boldsymbol{x})||P(\boldsymbol{\mathcal{E}})) &= \frac{1}{2}[\log\frac{|I|}{|\Sigma|} - h + tr\{I^{-1}\Sigma\} + (\vec{0}-\mu)^T I^{-1}(\vec{0}-\mu)] \\ &= \frac{1}{2}[-\log|\Sigma| - h + tr\{\Sigma\} + \mu^T\mu] \\ &= \frac{1}{2}[-\log\prod_i \sigma_i^2 - h + \sum_i \sigma_i^2 + \sum_i \mu_i^2] \\ &= \frac{1}{2}[-\sum_i \log\sigma_i^2 - h + \sum_i \sigma_i^2 + \sum_i \mu_i^2]. \end{aligned} \tag{S2}$$

REFERENCES

Duchi, J. (2007). Derivations for linear algebra and optimization. Technical report, Standford University.

Goldman, M. *et al.* (2019). The UCSC Xena platform for public and private cancer genomics data visualization and interpretation. bioRxiv; doi:10.1101/326470.

Hutter, C. and Zenklusen, J. C. (2018). The Cancer Genome Atlas: Creating Lasting Value beyond Its Data. *Cell*, **173**(2), 283–285.
